# The prevalence, relative risk factors and MTHFR C677T genotype of H type hypertension of the elderly hypertensives in Shanghai, China: a cross-section study: Prevalence of H type hypertension

**DOI:** 10.1186/s12872-021-02151-x

**Published:** 2021-08-04

**Authors:** Xiao-lin Qian, Hong Cao, Jun Zhang, Zhi-hui Gu, Wei-qin Tang, Lei Shen, Jia-lu Hu, Zhi-feng Yao, Lei Zhang, Min-na Tang, Xu-cheng Lv, Jun Zhou, Xue-juan Jin, Bin Hong, Zhao-qiang Cui, Jun-bo Ge

**Affiliations:** 1 Department of Cardiology, QingPu Branch of Zhongshan Hospital Affiliated to Fudan University, QingPu District Central Hospital Shanghai, Shanghai, China; 2 Department of Cardiology, QingPu District Jinze Community Health Center, Shanghai, China; 3 Department of Cardiology, QingPu District Xujing Community Health Center, Shanghai, China; 4 Department of Cardiology, QingPu District Zhujiajiao Community Health Center, Shanghai, China; 5 Department of Cardiology, QingPu District Xianghuaqiao Community Health Center, Shanghai, China; 6 Department of Cardiology, QingPu District Yingpu Community Health Center, Shanghai, China; 7 Department of Cardiology, Shanghai Institute of Cardiovascular Disease and Zhongshan Hospital Fudan University, Shanghai, China; 8 Department of Cardiology, Shanghai Zhujiajiao People’s Hospital, Shanghai, China

**Keywords:** H type hypertension, Hyperhomocysteinemia, MTHFR C677T, Prevalence, Risk factors

## Abstract

**Background:**

H type hypertension is defined as homocysteine (Hcy) ≥ 10 μmol/L in combination with primary hypertension. Studies demonstrated that the existence of hyperhomocysteine (HHcy) in hypertensive exacerbates the poor outcome of cardiocerebral incidents. This study was to investigate the current epidemic situation of H type hypertension and determine the risk factors in order to find intervention targets for H type hypertensives.

**Methods:**

We conducted a cross-sectional study using cluster sampling design in Shanghai, China from July 2019 and April 2020. 23,652 patients with primary hypertension were enrolled in this study. Their medical information was recorded, and the level of Hcy concentrations and methylenetetrahydrofolate reductase (MTHFR) C677T polymorphisms were detected.

**Results:**

In total, 22,731 of 23,652 patients were recorded. The mean age was 68.9 ± 8.6 y and 43% were men. 80.0% of the enrolled patients had H type hypertension. The frequency of allele T was 40.9%, and the proportions of the CC, CT, and TT genotypes were 36.1%, 46.0%, and 17.9%, respectively. Compared with the TT genotype, the plasma Hcy concentration levels were lower in patients with the CC/CT genotype (18.96 ± 13.48 μmol/L vs. 13.62 ± 5.20/14.28 ± 5.36, F = 75.04, *p* < 0.01). The risk for H type hypertension was higher in elderly people. Men had ~ 5.55-fold odds of H type hypertension compared with women. Patients with CT genotype and TT genotype had ~ 1.36- and ~ 2.76-fold odds of H type hypertension compared with those with CC genotype, respectively. Smoking and diabetes were not significantly associated with H type hypertension.

**Conclusions:**

The prevalence of H type hypertension in patients with primary hypertension was 80.0%, which was higher than the 75% found in prior report in China. Age, gender, and MTHFR C677T polymorphisms rather than smoking and diabetes were independently associated with H type hypertension.

## Background

Homocysteine (Hcy), a sulfur-containing amino acid, was deemed to participate in the process of arteriosclerosis as early as 1969 [1]. Since that time, multiple studies have indicated a positive relation between Hcy and stroke, coronary heart disease, peripheral artery disease, and hypertension [2–8]. An investigation using cross-sectional data from the Third National Health and Nutrition Examination Survey indicated that a 5-μmol/L-higher Hcy level was associated with increases in the diastolic and systolic blood pressure of 0.5 and 0.7 mmHg, respectively, 0.7 mmHg in men and 1.2 mmHg in women [8]. Liu et al. found that Hcy was an independent predictor of major adverse cardiac events in patients with acute coronary syndrome (ACS). Hcy was found to be significantly associated with the Global Registry of Acute Coronary Events (GRACE) risk score suggested by the research of 361 patients with ACS and a median follow-up of 43.3 months [9]. In a study consisting of 1823 stroke patients, after a follow-up of a median of 4.5 years, patients with high Hcy concentrations were associated with an increased risk of 1.74-fold for stroke recurrence, and 1.75-fold for all-cause mortality when compared to a low concentration group [4].

Hyperhomocysteinemia (HHcy) is common in the general population of Western countries as well as Eastern countries, particularly in patients with kidney dysfunction and vitamin defects [10–14]. The normal fasting plasma Hcy level is currently reported as between 5 and 15 μmol/L and includes free, disulfide-linked, and protein-bound Hcy [15]. However, a study prospectively investigating the relation between plasma Hcy levels and mortality showed that compared with patients with Hcy levels below 9 μmol/L, the mortality ratio was 1.9 for patients with Hcy levels of 9.0 to 14.9 μmol/L [16]. Similar results were shown in other reports. [17] H type hypertension is defined as hypertension with plasma Hcy levels over 10 μmol/L [18, 19]. In China, the proportion of H type hypertension in patients with primary hypertension was as high as 75% [2, 18]. Numerous studies demonstrated that the coexistence of hypertension and HHcy could exaggerate the adverse effect on cerebrovascular and cardiovascular diseases. An investigation including 17,601 persons who participated in the National Health and Nutrition Examination Survey in the USA revealed that men with hypertension without elevated Hcy were 5.4-fold as likely to suffer a prevalent stroke than those without either status; men with elevated Hcy without hypertension were 2.2-fold more likely to suffer a prevalent stroke as those without either condition; however, the presence of both hypertension and HHcy in men induced a 12.0-times higher odds of stroke. The trends in women were more severe [7].

Methylenetetrahydrofolate reductase (MTHFR) is a primary regulatory enzyme in the Hcy and folate metabolisms, catalyzing the reduction of 5,10-methyleneterahydrofolate to 5-methyleterahydrofolate [20, 21]. The common C677T polymorphism in the MTHFR gene converts alanine to a valine residue, decreasing its enzymatic activity and resulting in high Hcy and low folate levels in the blood [22]. Multiple studies confirmed that participants with the TT genotype had higher plasma Hcy levels than patients with the CC and CT genotype [23–25]. The objective of our study was to investigate the current epidemic situation of H type hypertension and determine the risk factors in order to find intervention targets for H type hypertensives.

## Methods

### Study population

The trial was a multisite cross-section population-based research with cluster sampling method. The design method was used because it provides a snapshot of the exposed variables across a wide population without manipulating or influencing the study population in any way. Subjects were classified according to their residential quarters from six towns of Qingpu district, the suburb of Shanghai city. (Yingpu town, Xiayang town, Xianghuaqiao town, Jinze town, Xujing town, and Zhujiajiao town). In total, there were 173 clusters being chosen from the six towns, including 18 clusters from Yingpu town, 42 clusters form Xiayang town, 12 clusters from Xianghuaqiao town, 47 clusters from Jinze town, 28 clusters from Xujing town, and 26 clusters from Zhujiajiao town. Subjects in accord with inclusion criteria were recruited. In total, 23,652 participants were recruited between July 2019 and April 2020 from Qingpu district. After excluding invalid information and abnormal data, 22,731 participants were included in the study.

The study protocol was approved by the ethics committee of Zhongshan Hospital Fudan University (Approval No: B2018-009B). All methods were performed in accordance with the relevant guideline and regulations. All invited participants provided written informed consent. The inclusion criteria were (1) age ≥ 18 years old; (2) confirmed with primary hypertension; and (3) no history of stroke. The exclusion criteria were (1) refusal to take part in the trial; and (2) refusal to provide a personal medical history.

### Data collection

The baseline demographic data, a medical history, and a physical and laboratory examination were collected at community health centers by trained research staff during the study entry process. Using a face-to-face interview, we collected information, including the name, gender, date of birth, history of smoking, medical history of diabetes mellitus, and hypertension and medication use. A history of hypertension was defined as patients who had hypertension prior to admission to the community health center, according to the question “Have you ever received a diagnosis of hypertension from any doctor or hospital?”; the use of antihypertension medication; or a systolic blood pressure ≥ 140 mmHg and/or diastolic blood pressure ≥ 90 mmHg. Diabetes mellitus was defined by a self-reported physician diagnosis, a self-reported current medical therapy, or a fasting blood-glucose ≥ 7 mmol/L. Smoking was defined by a self-reported history of smoking 1 cigarettes per day over at least 6 months. The blood samples were taken from all enrolled subjects for the test of the plasma Hcy level and only participants from the Xiayang community health center and Yingpu community health center were tested for the MTHFR C677T genotype. Patients whose plasma Hcy levels over 10 μmol/L combined with hypertension were defined as H type hypertension. After collection of data, it was edited, coded and examined for errors and omissions then corrected appropriately. All missing data were excluded after scrutiny before statistical analysis.

### Biochemistry analysis

Venous blood was drawn and collected at each community health center and allowed to stand for < 1 h and, then, centrifuged at 3000 × *g* for 10–15 min. The plasma and red blood cells were separated and frozen at − 80 °C, and stored for at most 2 weeks. All specimens were transported at 1–2 °C conditions by 1–2 day air transportation to a national qualified laboratory at Shenzhen Tailede enterprise for the test of the plasma concentration of Hcy and the MTHFR C677T genotype (CC, CT, and TT).

### Statistical analysis

Using IBM SPSS 20.0 for the primary analysis, we calculated the mean and standard deviation (SD) of the patients’ age and Hcy concentration. Student *t* tests were used to compare the mean values of the Hcy concentration between the two genders. Anova analysis was used to compare the mean values of the Hcy concentration among the CC, CT and TT genotypes. Plasma Hcy concentrations were divided into four groups (Hcy < 10 μmol/L, 10 μmol/L ≤ Hcy < 15 μmol/L, 15 ≤ Hcy < 20 μmol/L, and Hcy ≥ 20 μmol/L). The χ^2^ tests were used to compare the genotype proportion among different Hcy concentration groups. Univariate and multivariate logistic regression models were used to assess the association between H type hypertension and potential risk factors (age, gender, community health center, genotype, smoking, and diabetes). The odds ratios (ORs) and 95% confidence intervals (95% CIs) were used to evaluate the risk of H type hypertension after adjusting for important confounding factors. All *p* values were 2-tailed, and a significance level of 0.05 was used.

## Results

### Baseline characteristics

In total, 22,731 of 23,652 participants were recorded after eliminating missing data. For these 22,731 participants, 87.2% of them were over 60 years old, the mean age was 68.9 ± 8.6 years old and 43% were men. Patients with diabetes accounted for 31.0% (7046/22,731), and 32.4% (7371/22,731) were smokers. Male smokers accounted for 72.5% (7086/9767) of the men participants, whereas only 2.2% (285/12,964) female subjects had a habit of smoking. 2965 participants from Xiayang community health center and Yingpu community health center were recruited for the test of MTHFR C677T genotype at first, however only 2296 subjects were finally counted after excluding invalid data.

### Plasma Hcy concentration levels of patients with hypertension

The proportion of H type hypertension was 80.0% (18,151/22,731) in our study. We found that 31.5% patients had plasma Hcy concentrations of ≥ 15 μmol/L and the proportion of men was higher than that of women (χ^2^ = 1696, *p* < 0.01). 11.6% participants had plasma Hcy concentrations of ≥ 20 μmol/L, and men accounted for a larger percentage than women (χ^2^ = 787, *p* < 0.01). The proportions of patients with H type hypertension in the Yingpu, Jinze, Xiayang, Xianghuaqiao, Xujing, and Zhujiajiao community health centers were 64.1%, 82.3%, 67.8%, 78.9%, 92.6%, and 80.2%, respectively. The mean plasma Hcy concentration was 14.72 ± 8.37 μmol/L, and men had higher Hcy concentrations than women (17.13 ± 10.32 μmol/L vs. 12.90 ± 5.91 μmol/L, t = 38.96, *p* < 0.01.) The demographic characteristics of H type hypertension in our study are shown in Table 1.

**Table 1:** The demographic data and clinical characteristics of patients with H type hypertension

	Men			Women		
	H type hypertension/total (n/n)	Hcy (μmol/L) mean ± SD	The ratio of H type hypertension (%)	H type hypertension /total (n/n)	Hcy (μmol/L) mean ± SD	The ratio of H type hypertension (%)
23–29 years old	1/1	11.89	100.0	1/2	11.02 ± 3.43	50.0
30–39 years old	19/19	15.85 ± 9.55	100.0	2/14	8.20 ± 1.72	14.3
40–49 years old	84/109	14.69 ± 9.18	77.1	43/150	9.65 ± 3.75	28.7
50–59 years old	757/935	13.99 ± 6.77	81.0	696/1687	10.20 ± 3.21	41.3
60–69 years old	3661/4062	15.62 ± 8.92	90.1	3324/5250	11.67 ± 4.35	63.3
70–79 years old	3402/3546	18.16 ± 10.78	95.9	3672/4365	14.01 ± 6.41	84.1
≥ 80 years old	1079/1095	22.34 ± 13.47	98.5	1410/1496	17.25 ± 8.10	94.3
Total	9003/9767	17.13 ± 10.32	92.2	9148/12,964	12.90 ± 5.91	70.6
Community health center						
Yingpu	342/404	14.87 ± 8.49	84.7	443/821	11.38 ± 4.93	54.0
Jinze	2377/2563	17.58 ± 11.41	92.7	2458/3313	13.30 ± 5.60	74.2
Xiayang	1262/1433	15.55 ± 9.76	88.1	1405/2503	11.38 ± 4.40	56.1
Xianghuaqiao	1520/1706	16.13 ± 8.91	89.1	1402/1996	12.75 ± 5.73	70.2
Xujing	1953/1985	19.20 ± 11.03	98.4	2022/2308	14.83 ± 7.45	87.6
Zhujiajiao	1549/1676	16.89 ± 9.37	92.4	1418/2023	12.69 ± 5.88	70.1
Smoking	6427/7086	16.58 ± 10.33	90.7	202/285	13.65 ± 6.56	70.8
Diabetes	2111/2318	16.93 ± 10.87	91.1	1434/2163	12.48 ± 5.62	66.3

### MTHFR C677T genotype of patients with hypertension

**Fig. 1:**
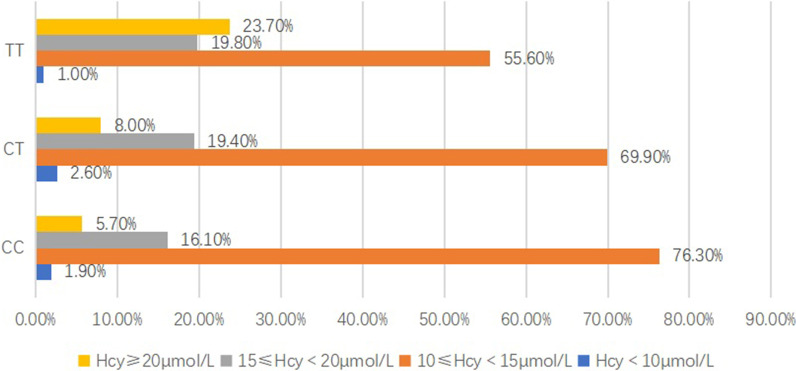
Distribution of the plasma homocysteine concentration level groups by methylenetetrahydrofolate reductase genotype for participants

**Fig. 2:**
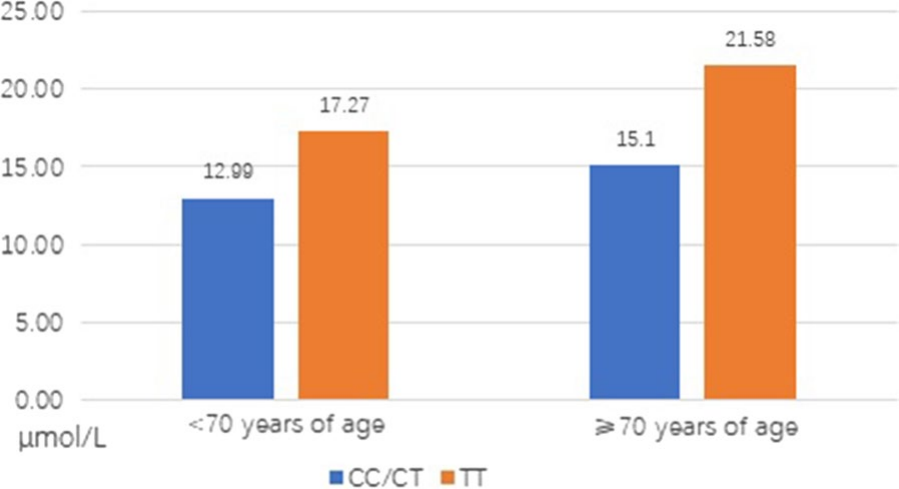
The plasma homocysteine concentration levels by age and methylenetetrahydrofolate reductase genotypes of h type hypertension

Of the 2296 patients tested for the MTHFR C677T gene type, the mean age was 69.0 ± 8.1 years old, the frequency of allele T was 40.9% and the proportions of the CC, CT, and TT gene types were 36.1%, 46.0%, and 17.9%, respectively. The MTHFR C677T genotype distribution was different from the frequencies predicted by Hardy–Weinberg equilibrium. Compared with the TT genotype, the plasma Hcy concentration levels were lower in patients with the CC/CT genotype (18.96 ± 13.48 μmol/L vs.13.62 ± 5.20/14.28 ± 5.36, F = 75.04, *p* < 0.01) (Table 2). In the Hcy ≥ 20 μmol/L group, the proportions of the CC, CT, and TT genotype were 5.7%, 8.0%, and 23.7%, respectively, while in the 10 μmol/L ≤ Hcy < 15 μmol/L group, the ratios were 76.3%, 69.9%, and 55.6%, respectively (Fig. 1). The distribution of the plasma Hcy concentration level groups by C677T genotypes was significantly different (χ^2^ = 120.73, *p* < 0.01). Compared with the ≥ 70 years of age group, the mean Hcy concentration level was 21.58 μmol/L in subjects with the TT genotype, which was much higher than that of the < 70 years of age group, whose mean Hcy concentration level was only 17.27 μmol/L. However, the difference was not as clear in subjects with the CC/CT genotype (Fig. 2).

**Table 2:** The demographic characteristics of patients with different methylenetetrahydrofolate reductase gene types

	CC	CT	TT	χ^2^*/F*	*p* value
N (%)	828 (36.1)	1058 (46.0)	410 (17.9)	NA	NA
Men (%)	411 (49.6)	488 (46.1)	198 (48.3)	2.35	0.31
Age, mean ± SD, y	69.46 ± 8.19	69.14 ± 8.18	67.77 ± 7.55	6.205	< 0.01
Smoking, n, (%)	142 (17.1)	214 (20.2)	84 (20.5)	3.404	0.18
Diabetes, n, (%)	144 (17.4)	179 (16.9)	62 (15.1)	1.04	0.59
Hcy, μmol/L mean ± SD	13.62 ± 5.20	14.28 ± 5.63	18.96 ± 13.48	75.04	< 0.01

The univariate logistic regression analysis showed that the differences of gender, age, cigarette use, and community health center in H type hypertension and non-H type hypertension patients were significant. Men had ~ 4.91-fold odds of H type hypertension compared with women (95% CI 4.52, 5.33, *p* < 0.01). With age, area, smoking, and diabetes included in the logistic regression model, men had ~ 5.55-fold odds of H type hypertension compared with women (95% CI 4.74, 6.50, *p* < 0.01). For age, the analysis demonstrated that, after age 40, the risk of H type hypertension increased as people grew older, and the risk remained unchanged when including gender, area, smoking, and diabetes in the multivariate logistic regression model (Table 3).

**Table 3:** Univariate and multivariate logistic regression analysis of H type hypertension. odds ratio (OR) and 95% confidence interval (95% CI)

	Univariate logistic regression analysis			Multivariate logistic regression analysis*		
	OR	95% CI	*p*	OR	95% CI	*p* value
Gender						
Men	4.91	4.52, 5.33	< 0.01	5.55	4.74, 6.50	< 0.01
Women	1.00			1.00		
Age						
< 30 years old	0.08	0.01, 0.91	0.04	0.03	0.001, 0.62	0.02
30–39 years old	0.07	0.03, 0.15	< 0.01	0.05	0.02, 0.13	< 0.01
40–49 years old	0.04	0.03, 0.05	< 0.01	0.04	0.02, 0.05	< 0.01
50–59 years old	0.05	0.04, 0.06	< 0.01	0.05	0.04, 0.06	< 0.01
60–69 years old	0.12	0.10, 0.15	< 0.01	0.10	0.08, 0.13	< 0.01
70–79 years old	0.35	0.28, 0.43	< 0.01	0.28	0.22, 0.37	< 0.01
≥ 80 years old	1.00			1.00		
Community health center						
Yingpu	1.00			1.00		
Jinze	2.58	2.26, 2.96	< 0.01	2.31	1.99, 2.70	< 0.01
Xiayang	1.17	1.02, 1.34	0.02	1.32	1.14, 1.54	< 0.01
Xianghuaqiao	2.09	1.81, 2.40	< 0.01	1.72	1.46, 2.02	< 0.01
Xujing	6.96	5.91, 8.20	< 0.01	3.19	2.47, 4.11	< 0.01
Zhujiajiao	2.26	1.96, 2.60	< 0.01	2.15	1.82, 2.56	< 0.01
Smoking	3.67	3.28, 4.04	< 0.01	0.94	0.80, 1.12	0.50
Diabetes mellitus	1.02	0.94, 1.10	0.70	0.97	0.88, 1.06	0.44

^*^Adjusted for gender, age, area, smoking, and diabetes

The risk of having H type hypertension was also different in different community health centers. Subjects selected from the Xujing community health center, which had the highest prevalence of H type hypertension, had ~ 6.96-fold odds of H type hypertension than those from the Yingpu community health center (95% CI 5.91, 8.20, *p* < 0.01), which had lowest prevalence of H type hypertension. The regional differences persisted after controlling for gender, age, smoking, and diabetes. In addition, cigarette smokers had ~ 3.67-fold odds of H type hypertension compared with non-smokers (95% CI 3.28, 4.04, *p* < 0.01). However, in a multivariate model with further control for age, gender, area, and diabetes, this association was attenuated toward null. Diabetes was not associated with H type hypertension in either the univariate or multivariate analysis (Table 3).

Of all subjects tested for the C677T gene type, compared with patients with the CC genotype, patients with the CT gene type and TT gene type had ~ 1.36- and ~ 2.76-fold higher odds of H type hypertension, respectively. The association remained unchanged after controlling for the ln-age and gender in the multivariate logistic regression model (Table 4).

**Table 4:** The OR and 95% CI of H type hypertension by the methylenetetrahydrofolate reductase gene type, age, and gender

	OR (95% CI)	*p* value	OR * (95% CI)	*p* value
Gene type				
CC	1.00		1.00	
CT	1.36 (1.10, 1.68)	< 0.01	1.53 (1.22, 1.92)	< 0.01
TT	2.76 (2.14, 3.57)	< 0.01	3.77 (2.85, 4.99)	< 0.01
ln-age	159.77 (66.61, 383.22)	< 0.01	391.32 (152.61, 1003.41)	< 0.01
Gender	2.55 (2.11, 3.07)	< 0.01	2.92 (2.39, 3.57)	< 0.01

^*^Adjusted for gene type, ln-age, and gender

## Discussions

It was reported that 23.2% of Chinese adult population ≥ 18 years of age had hypertension, and the prevalence of hypertension in Shanghai city was 29.1%, which ranked in the third place, after Beijing city and Tianjin city [26]. The research revealed that in Shanghai, the prevalence rate of hypertension in urban was 28.7%, which was significantly lower than that in rural region, whose prevalence rate was 39.2%. However, the differences of prevalence rate between male and female were not significant [26]. Our research was carried out in Qingpu district, the suburb of Shanghai. Unlike the distribution of hypertension between male and female in Shanghai, in our study, the ratio of H type hypertension was higher in male, when compared with female.

To our knowledge, the prevalence of H type hypertension was 75% in China as reported in a previous study, which was conducted in six main cities of China [18]. However, after investigating the 22,731 participants in our study, the prevalence of H type hypertension was 80.0%, and patients with a Hcy concentration ≥ 15 μmol/L occupied 31.5%.

The high prevalence of H type hypertension in our study may be caused by the high proportion of the aged. The mean age of all subjects was 68.9 ± 8.6 years old, and up to 87.2% of them were over 60 years old. In general, the risk of having H type hypertension is positively associated with age. Compared with subjects ≥ 80 years old, subjects at ~ 40 y, ~ 50 y, ~ 60 y, and ~ 70 y of age had only ~ 0.04-, ~ 0.05-, ~ 0.12-, and ~ 0.35-fold odds of H type hypertension, respectively, which showed an upward trend. After controlling gender, area, and other factors, this trend did not disappear. The same results were also confirmed in other studies. In a study including 810 individuals aged 65–74 years from the north areas of China, the prevalence of HHcy (Hcy ≥ 16 μmol/L) was 51.6% [27].

Another reason may be the changing lifestyles and dietary habits that resulted from the rapid industrialization in China, particularly in Shanghai, which is an international first-tier city. A systematic review, comprising 36 studies, explicated that the prevalence of HHcy (> 15 μmol/L) increased from 22.7% in 1990–2005 to 29.6% in 2006–2012 in Chinese people [28]. Another article suggested that individuals living in urban areas had significantly higher median Hcy concentrations than those living in rural areas [13]. Surveys conducted in Beijing city, the capital of China, implicated similar results [29, 30]. Zhang et al. disclosed that the proportion of HHcy (> 15 μmol/L) in patients with hypertension was 35.3%, similar to our study (31.5%), after investigating 866 citizens in Beijing. In his study, the mean age was 69.5 ± 8.1 years old and men accounted for 45%, which was in accordance with our study in demographic characteristics [29].

We suggest that factors not captured by our study might contribute to the high prevalence of H type hypertension, eg, impaired renal function. In China, the prevalence of hypertension among predialysis chronic kidney disease (CKD) was 78.4% [31], and the prevalence of hypertension among hemodialysis patients was as high as 70–90% [32]. In addition, there is an inverse correlation between an elevation of plasma Hcy and the development of CKD in a general population [33]. The overall prevalence of CKD in Shanghai was 16% [34], which was higher than the average prevalence rate of CKD (10.8%) in China [35]. Thus, the high prevalence of CKD in Shanghai may increase the chance of suffering H type hypertension.

Men had higher Hcy concentrations than women (17.13 ± 10.32 μmol/L vs. 12.90 ± 5.91 μmol/L), and, compared to women, men had ~ 4.91-fold odds of H type hypertension, which was in agreement with previous results [8, 13, 14, 21, 28]. Previous observations indicated that estrogen was inversely related to Hcy concentrations [36, 37], and Hcy was positively associated with postmenopausal status (defined as age ≥ 55 years) [8]. The reason why men had significantly higher Hcy concentrations than women at each age group was likely that men had lager muscle mass (as 75% of plasma Hcy is made in conjunction with creatine-creatinine synthesis) [38].

Previous study demonstrated that the smoking rate was higher in male compared with female (58% vs 5%) [39]. In our study, the gap was wider (72.5% for men and 2.2% for women). Herein, cigarette smokers had ~ 3.67-fold odds of H type hypertension compared with non-smokers. Nevertheless, the difference attenuated when adjusting for gender, which suggested that the difference in gender was the main cause for the cigarette use difference of Hcy concentrations in the study population.

The prevalence of H type hypertension varied in different community health centers, and the difference remained significant after adjusting for gender, age, smoking, and diabetes. This may result from the uneven daily dose of folic acid and vitamin B-12 prescribed to the patients in different community health centers. However, more information would be required to prove this.

Although 91.1% of male diabetic subjects had H type hypertension in our study, diabetes was not associated with H type hypertension in either the univariate or multivariate analysis. There may be some reasons accounting for the contradiction. As demonstrated before, men had higher risk of suffering H type hypertension than women, which inducing the high proportion of H type hypertension in diabetic subjects. Other mechanisms, eg, insulin resistance and/or impaired renal function, may also be responsible for the high blood pressure values observed in diabetic subjects. Previous research revealed that diabetes-related variables, eg, insulin resistance, metabolic control and oral hypoglycemic drugs, may specifically modulate the concentration of Hcy [40–42]. Likewise, Russo et al. addressed that mild hyperhomocysteinemia and the MTHFR TT mutation do not predict the incidence of macrovascular complications in type 2 diabetic patients [43].

Overall, of the 2296 patients tested for the MTHFR C677T gene type, the frequency of allele T was 40.9% and the proportions of the CC, CT, and TT gene types were 36.1%, 46.0%, and 17.9%, respectively. McAndrew et al. analyzed 101 unselected Caucasians and 102 unselected African Americans and reported that the frequency of allele T was 30% in Caucasians and 10% in African Americans [44]. Pollak et al. studied the frequency of the C677T mutation in healthy Israeli ethnic groups and found that the frequencies of allele T were 44% in Ashkenazi Jews, 33% in Muslim Arabs, 36% in North Africans, 39% in Libyans, 36% in Moroccans, 24% in Iraqis, 29% in Persians, 31% in Sephardic Jews, and 19% in Yemenites [45]. With respect to the Asian population, an article finished in Japan disclosed that the frequency of the T allele was 33% in healthy Japanese men [24].

The plasma Hcy concentration level was significantly higher in subjects with the TT genotype compared with those with the CC/CT genotype (18.96 ± 13.48 μmol/L vs.13.62 ± 5.20/14.28 ± 5.36, F = 75.04, *p* < 0.01). In the group with high Hcy concentration (> 20 μmol/L), the proportion of the TT genotype was much higher compared with the CC genotype (23.70% vs. 5.70%), while, in the lower Hcy concentration group (10 μmol/L ≤ Hcy < 15 μmol/L), the result was the opposite. The ratio of the CC genotype was higher than the ratio of the TT genotype (76.30% vs. 55.60%).

The Hcy concentration levels were much higher in elders with the TT genotype. The average Hcy concentration in participants < 70 years of age with the CC/CT genotype was only 12.99 μmol/L; however, in participants < 70 years of age with the TT genotype, the mean Hcy concentration was 17.27 μmol/L, which was lower compared with participants > 70 years of age with the TT genotype whose mean Hcy concentration was 21.58 μmol/L. Our results are consistent with previous research [24, 46, 47]. An article revealed that plasma Hcy concentrations were significantly higher in patients with the TT genotype compared with patients with the CC/CT genotype (16.4 ± 6.2 μmol/L vs. 14.5 ± 3.6 μmol/L) [24]. A meta-analysis including 36 studies demonstrated that those bearing the TT gene type had higher plasma Hcy concentrations of 2.6 μmol/L (25%) than those with the CC gene type. This study also indicated that the TT gene type was associated with elevated mean Hcy concentration levels in the likely well-nourished groups of American, Canadian, Dutch, Norwegian, Italian, and Irish subjects and that high Hcy concentration levels were confined primarily to those of the TT gene type with folate levels below the median or in the lowest quartile of serum or plasma folate [47].

Of all the participants tested for the C677T gene type, compared with patients with the CC genotype, patients with the CT gene type and TT gene type had ~ 1.36- and ~ 2.76-fold higher odds of H type hypertension, respectively. After controlling for the ln-age and gender, the association remained unchanged. Yuan et al. selected 224 cases of ischemic stroke and H type hypertension and randomly divided them into two groups with and without treatment of mecobalamin to analyze the effect of mecobalamin on the early-functional outcomes of patients with ischemic stroke and H type hypertension. The results showed that mecobalamin could reduce the level of plasma Hcy, resulting in a more significant functional recovery [48]. Another investigation suggested that the folate and vitamin B-12 levels were lower among those with the TT gene type compared with those with the CC/CT gene type and indicated that those with the TT genotype may have both folate and vitamin B-12 insufficiencies as well as decreased MTHFR enzyme activity [25]. Jacques et al. revealed that subjects with the TT genotype may have higher folate requirements to regulate Hcy, particularly if they also have a folate deficiency [49]. As a result, it is necessary for H type hypertension patients with the TT genotype to begin folate and vitamin B-12 therapy as early as possible.

## Limitations

Our study had certain limitations. Parts of our information depended on self-reports of medical history, including hypertension and diabetes. The age of our participants mostly ranged from 50 to 90 years old, leading to an insufficient sample size in participants whose age ranged from 20 to 50 years old. This inevitably caused bias in the explanation of patients from 20 to 50 years old. In addition, the study was cross-sectional, limiting the interpretation of cause and effect of any associations found. Besides, we haven’t taken the folic acid and vitamin B-12 status into consideration, which could also influence the Hcy concentrations.

## Conclusions

The prevalence of H type hypertension in patients with primary hypertension was 80.0%, which was higher than the 75% found in prior report in China. Age, gender, and MTHFR C677T polymorphisms rather than smoking and diabetes were independently associated with H type hypertension. Increased attention should be paid to the prevention and treatment of H type hypertension in China.

## Data Availability

The datasets used during the current study are available from the corresponding author on reasonable request.

## Abbreviations

Hcy: Homocysteine
HHcy: Hyperhomocysteine
ACS: Acute coronary syndrome
GRACE: Global Registry of Acute Coronary Events
MTHFR: Methylenetetrahydrofolate reductase
CKD: Chronic kidney disease
